# Provocation of dry eye disease symptoms during COVID-19 lockdown

**DOI:** 10.1038/s41598-021-03887-4

**Published:** 2021-12-24

**Authors:** Nutnicha Neti, Pinnita Prabhasawat, Chareenun Chirapapaisan, Panotsom Ngowyutagon

**Affiliations:** Department of Ophthalmology, Faculty of Medicine Siriraj Hospital, Mahidol University, 2 Wanglang Rd., Siriraj District, Bangkok Noi, 10700 Bangkok Thailand

**Keywords:** Corneal diseases, SARS-CoV-2, Risk factors

## Abstract

To assess the impact of COVID-19 lockdown measures on dry-eye symptoms in a community-based population, a cross-sectional study was conducted during the first wave of the COVID-19 outbreak in Thailand. An online survey was distributed via social media between June and July 2020. The questionnaire elicited information on demographics, dry-eye symptoms, use of visual display terminals, and mental health status. There were 535 respondents. Thirty-seven percent reported having been diagnosed with dry-eye disease (DED). During the lockdown, the mean dry-eye symptom score (DESS) of overall participants dropped significantly from 81.6 ± 15.9 to 79.8 ± 17.4 (*P* < 0.001). The mean, daily, visual display terminal (VDT) usage increased from 10.55 ± 5.16 to 13.08 ± 5.65 h (*P* < 0.001). A negative correlation between age and VDT usage was observed in both the normal and lockdown situations. One-quarter of all participants had an abnormal mental health status. The female gender (OR 1.86; 95% CI 1.14–3.04) and increased VDT usage during the lockdown (OR 5.68; 95% CI 3.49–9.23) were independently associated with worsening dry-eye symptoms. The lockdown measures abruptly altered the behaviors and lifestyles of the overall population. Excessive exposure to VDTs were associated with deteriorated dry-eye symptoms, and it possibly contributed to the increased DED incidence in the surveyed population.

## Introduction

The outbreak of coronavirus disease 2019 (COVID-19) had a massive impact on global health systems^1^. In March 2020, COVID-19 was declared a pandemic disease by the World Health Organization. The disease is highly contagious in aerosol form and on contaminated surfaces^2^. As of July 2021, there had been 179.9 million cases in 222 countries, with 3.9 million mortalities. To curb the spread of COVID-19, numerous countries, including Thailand, imposed extraordinary measures, such as lockdowns, flight restrictions, social distancing, working from home, and department-store closures.

The COVID-19 lockdown measures enforced during 2020 had a major impact on the lifestyles and viewing habits of the world population. People were reported to be spending more time indoors, in some cases, up to 20–24 h per day^3^. Students and company employees were forced to study, work, and attend meetings online. The use of visual display terminals (VDTs), which include computer screens, tablets and smartphones, rose significantly, whereas the undertaking of physical activities declined^4–6^. While focusing on VDTs, a longer blinking interval exacerbates the evaporation of tears and eventually increases the risk of developing dry-eye disease (DED)^7–10^.

Moreover, the pandemic outbreak along with self-isolation measures impaired the overall population’s mental health status^3^. Community-based adults spent excessive amounts of time following COVID-19 news updates, which was found to be positively associated with mental health problems such as acute stress, anxiety, and depression^11–14^. Moreover, an association between psychiatric disorders and DED was reported by several studies^15–20^.

In the past, when people used VDTs relatively infrequently, the prevalence of DED was reported to range from 5 to 50%, to increase with age, and to disproportionately affect women more than men^21–23^. However, the marked rise in VDT usage during the last decade has corresponds to a shift in the epidemiology of DED. The condition now affects younger ages as well as both genders equally^10,24^. DED symptoms include discomfort, grittiness, tearing, a burning sensation, and vision fluctuation. The main treatments are the administration of tear substitutes, secretagogues, and anti-inflammatory drugs^25^. DED was reported to negatively impact work productivity and the quality of life^26,27^. Moreover, the increasing number of cases of DED was estimated to have an enormous negative impact upon economies and healthcare systems across Asia, Europe, and the United States^28^.

The objective of this study was to assess the impact of the COVID-19 lockdown measures on dry-eye symptoms in the Thai population. The study also examined how the lockdown affected mental health and VDT-viewing habits in Thailand, and their associations with dry-eye symptoms.

## Materials and methods

This cross-sectional study was carried out online during the first COVID-19 lockdown period in Thailand (June to July 2020). The procedures followed the tenets of the Declaration of Helsinki. Before commencement of this research, its protocol was approved by the Committee for the Protection of Human Participants in Research, Faculty of Medicine, Siriraj Hospital (approval number: 517/2020). The work was registered at the Thai Clinical Trials Registry (TCTR20200709002).

The survey was conducted using an online survey portal, Google Forms. Responses were voluntary and anonymous. An online link targeting a community-based population was sent via social networks such as Facebook, Line, and WhatsApp. In all, 535 individuals responded. The survey questionnaire was divided into 4 sections: demographic data, dry-eye symptoms, VDT-viewing habits, and mental health.

The respondents who reported having been diagnosed with DED by an ophthalmologist were assigned to a “DED group” for analysis purposes. The remaining participants became the controls and were allotted to a “non-DED group”. The dry-eye-symptom portion of the study questionnaire was modified and translated from a symptom questionnaire developed by Kojima and associates in 2011^29^. The questionnaire was culturally adapted and translated into Thai by 3 qualified native-Thai cornea specialists proficient in spoken and written English (PP, CC, PN). To do this, the specialists made independent translations of the original English version, and the final Thai version was decided after discussion by the specialists. The scale's internal consistency was calculated using Cronbach's alpha coefficient (Cronbach's alpha = 0.912). The construct validity was evaluated by using the correlation between the total scores of a Thai version of the Dry Eye–Related Quality-of-Life Score (DEQS-Th) questionnaire^30^ and the dry-eye symptom score (DESS) of this study. Higher DESS scores indicate fewer dry-eye symptoms, whereas DEQS-Th scores indicate the opposite. The correlation coefficient between the total scores of the scales was − 0.805 (*P* < 0.001). The test–retest reliability showed a significant correlation between the first and second applications, with r = 0.921 (*P* < 0.001). The DESS consisted of 14 self-assessment questions addressing the common dry-eye-related ocular symptoms. They were dryness; irritation; grittiness; soreness; fatigue; eye strain; redness; itching; difficulty opening the eyelids; discomfort; drowsiness; and limitations while reading, operating a computer, or watching television. Each question was graded by frequency: no symptoms, 4 points; hardly ever, 3 points; sometimes, 2 points; often, 1 point; and always, 0 points. The final DESS was calculated by (the sum of scores for all answered questions × 100)/(number of answered questions × 4). The DESS ranged from 0 to 100, with higher scores signifying fewer dry-eye symptoms.

To evaluate the VDT-viewing habits of the participants, the average number of hours they were exposed to VDTs each day, by purpose, were asked. Averages were obtained for both the pre-lockdown (“normal”) and lockdown situations. The lockdown period was defined as the time between March 22, 2020 and the day the survey was completed. Four categories of VDT usage were identified: (1) work/study, (2) social media, (3) video logs/TV series/movies, and (4) news updates. The term “long-time VDT usage” was defined as a daily average of 6 or more hours exposure to VDTs^31^.

The Thai General Health Questionnaire-12 (Thai GHQ-12) was used to evaluate the mental health status of the respondents^32^. It contained 4 questions related to major mental health problems: anxiety, social impairment, unhappiness, and hypochondriasis. Each question assessed the severity of mental problems over the preceding few weeks. The total-score range was 0 to 12, with higher scores indicating worse conditions. A total score greater than or equal to 2 was considered to represent an abnormal mental health status.

### Statistical analysis

The data analyses were performed using PASW Statistics for Windows (version 18.0; SPSS Inc., Chicago, IL, USA). Categorical variables are reported as N (%), and continuous variables as mean (SD). Associations between variables were tested using univariate analysis (chi-squared test). Factors with *P* < 0.1 in the univariate analysis were entered in a multivariate logistic regression model to identify the variables contributing to the worsening of dry-eye symptoms during COVID-19. Spearman’s correlation was used to identify relationships between pairs of ordinal or continuous data. The odds ratios (OR) and corresponding 95% CI were used to determine strengths of association. A *P* value of < 0.05 was deemed statistically significant.

### Human rights statements and informed consent

This study was performed in accordance with the Declaration of Helsinki and approved by the ethical committee on human experimentation of Faculty of Medicine Siriraj Hospital, Thailand. Informed consent was obtained, by requiring all respondents to answer the acceptance checkbox, before being included in the study.

### Animal rights

This study did not associate with any animal subject.

## Results

**Figure 1:**
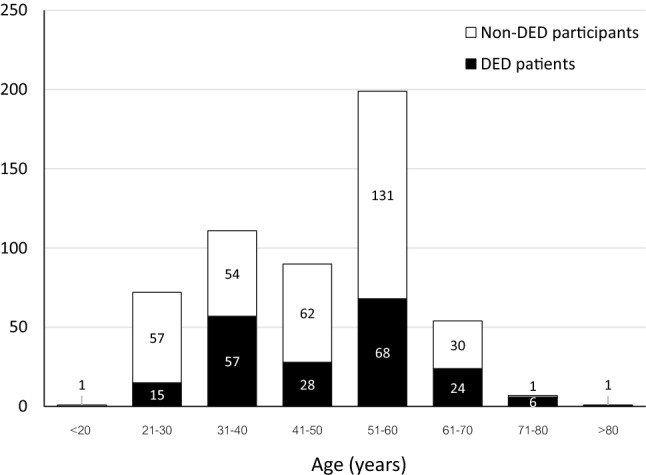
Distribution of ages of participants.

A total of 535 survey responses were recorded. The ages of the participants ranged from 18 to over 80 years, with the most frequent age range being 51–60 years (37.2%; Fig. 1). Sixty-seven percent (67.3%) were women. Thirty-seven percent (37.2%) of all participants reported they had been diagnosed with DED by an ophthalmologist. Concurrent ocular disorders were predominantly refractive error (73.2%), followed by vitreous degeneration (13.1%) and allergy (10.1%). Most DED patients used 1 or 2 types of dry-eye medication (88.9%), with 59.8% of the patients using only 1 type. The frequency of dry-eye medication usage was generally less than 4 times a day (90.9%), and 41.7% used it less than once daily.

**Figure 2:**
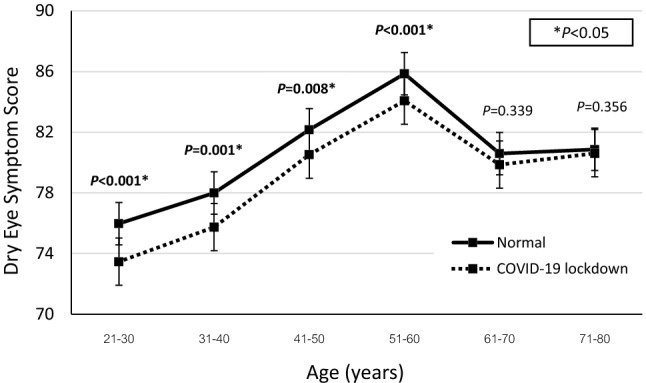
Dry-eye symptom scores (DESS) of each age group for normal and COVID-19 lockdown situations.

During the COVID-19 lockdown, the overall DESS dropped significantly from a mean of 81.6 ± 15.9 to 79.8 ± 17.4 (*P* < 0.001). The DESS also decreased significantly for the DED patients during the lockdown, from 74.1 ± 14.6 to 72.2 ± 16.0 (*P* < 0.001). Similarly, for the non-DED group, the DESS fell from 86.0 ± 15.0 to 84.3 ± 16.7 (*P* < 0.001). However, there was no statistical difference in the degrees by which the DESS of the DED and non-DED groups declined (*P* = 0.69). In terms of age, the DESS was lowest for the 21–30 age group (normal situation, 75.97 ± 17.62; during the lockdown, 73.46 ± 18.68; *P* < 0.001). The DESS was highest for the 51–60 age group (normal, 85.86 ± 13.41; lockdown, 84.08 ± 15.30; *P* < 0.001; Fig. 2). For the participants who were 60 years old or less, the DESS dropped significantly during the lockdown for both the DED and the control group (DED group, from 74.16 ± 13.94 to 71.96 ± 15.21, with *P* < 0.001; non-DED group, from 85.9 ± 15.36 to 84.05 ± 17.18, with *P* < 0.001). In the case of the respondents who were older than 60 years, there was a nonsignificant change in the DESS for both the DED and non-DED groups (DED group, from 73.8 ± 17.7 to 73.2 ± 19.9, with *P* = 0.64; non-DED group, from 87.0 ± 10.2 to 86.2 ± 10.9, with *P* = 0.17; Table 1). When the subjects experienced eye discomfort, 40.3% rested their eyes, 31.9% used artificial tears, 15.8% increased their eye-blinking rate, and 2.5% rinsed their eyes with tap water. During the lockdown, 10.6% of the DED patients used artificial tears more often while 8.6% used them less frequently; the rest maintained their normal frequency.

**Table 1:** Comparison of the mean dry-eye symptom scores of the dry-eye and non-dry-eye participants.

	Dry-eye symptom score		*P*
	Normal situation	COVID-19 lockdown	
	Mean (SD)	Mean (SD)	
**Dry-eye participants**			
Total	74.10 (14.55)	72.16 (15.98)	< 0.001*
Age ≤ 60 years	74.16 (13.94)	71.96 (15.21)	< 0.001*
Age > 60 years	73.79 (17.73)	73.21 (19.88)	0.639
**Non-dry-eye participants**			
Total	86.00 (14.95)	84.25 (16.70)	< 0.001*
Age ≤ 60 years	85.9 (15.36)	84.05 (17.18)	< 0.001*
Age > 60 years	86.98 (10.15)	86.23 (10.88)	0.167

*P < 0.05; higher dry-eye symptom score indicates fewer dry-eye symptoms.

**Figure 3:**
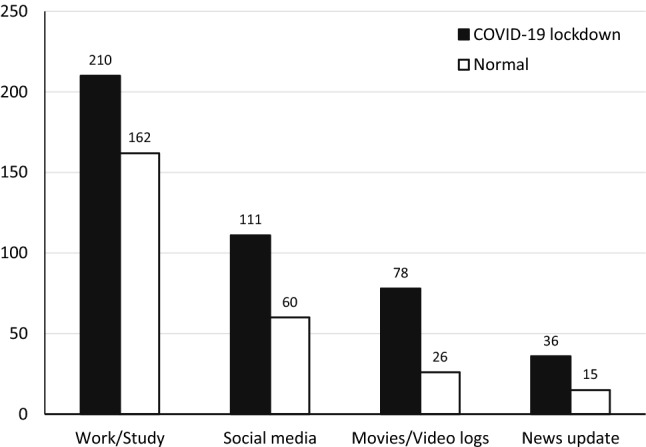
The number of participants using long-time visual display terminals, by purpose, during the normal and COVID-19 lockdown situations.

Daily VDT usage rose significantly during the lockdown, from a mean of 10.55 ± 5.16 h to 13.08 ± 5.65 h (*P* < 0.001). For the participants aged up to 60 years, the daily hours went up from 11.03 ± 5.17 to 13.74 ± 5.50 (*P* < 0.001). As to the participants older than 60 years, their average grew from 6.84 ± 3.32 to 8.05 ± 4.15 h (*P* < 0.001; Table 2). There was a negative correlation between age and VDT usage during both the normal and lockdown situations (normal, Spearman’s rho = − 0.386, with *P* < 0.001; lockdown, rho = -0.374, with *P* < 0.001). The prevalence of long-time VDT usage climbed for all purposes: news updates, watching movies and videos, social media surfing, and work/study (Fig. 3). In both the normal and lockdown situations, work/study was the major activity contributing to prolonged VDT usage. However, the greatest increase in the number of participants using VDTs for extended periods was demonstrated by those using VDTs for entertainment purposes (such as watching movies and videos).

**Table 2:** Visual display terminal usage of each age group during the normal and COVID-19 lockdown situations.

Age range	VDT usage (h)		*P*
	Normal situation	COVID-19 lockdown	
	Mean (SD)	Mean (SD)	
**Age ≤ 60 years**			
Total	11.03 (5.17)	13.74 (5.50)	< 0.001*
21–30	13.64 (4.59)	15.92 (4.96)	< 0.001*
31–40	12.29 (5.47)	14.69 (5.60)	< 0.001*
41–50	10.47 (5.03)	13.78 (5.09)	< 0.001*
51–60	9.61 (4.73)	12.36 (5.43)	< 0.001*
**Age > 60 years**			
Total	6.84 (3.32)	8.05 (4.15)	< 0.001*
61–70	6.84 (3.08)	8.15 (4.13)	< 0.001*
71–80	6.71 (5.28)	7.36 (4.87)	0.001*

*P < 0.05.

During the lockdown, one-quarter of all participants reported mental health problems (25.9%). The top-rated abnormalities were feeling constantly under strain, feeling unhappy, and not enjoying normal daily activities. The overall Thai GHQ-12 score during the lockdown showed nonsignificant differences between age groups (1.84 ± 2.65 for participants aged up to 60, vs 1.23 ± 2.09 for older respondents; *P* = 0.082); genders (1.69 ± 2.60 for males, vs 1.80 ± 2.59 for females; *P* = 0.650); and the presence of concurrent DED (1.71 ± 2.48 for the DED group, vs 1.79 ± 2.66 for the non-DED group; *P* = 0.727). Participants with a DESS of less than 75 were associated with having abnormal mental health during the lockdown (OR 1.91; 95% CI 1.28–2.84; Table 3). Actions to cope with the stress during the pandemic were buying personal protective equipment such as face masks (30.8%); following COVID-news updates (24.8%); exercising (24.4%); minimizing watching, reading, or listening to COVID-19 news (9.4%); meditation/praying (8.4%); and staying at home (2.1%).

**Table 3:** Risk factors for abnormal Thai GHQ-12 score during the lockdown.

	Thai GHQ-12 score; n (%)		*P*	OR	95% CI
	Normal	Abnormal			
Age ≤ 60 years	347 (87)	126 (92)	0.163	1.68	(0.85–3.33)
Female	264 (66)	96 (70)	0.461	1.19	(0.78–1.81)
DED patients	149 (37)	50 (36)	0.918	0.96	(0.64–1.44)
DESS < 75	118 (30)	61 (45)	0.002*	1.91	(1.28–2.84)

*P < 0.05; *DED* dry-eye disease, *DESS* dry-eye symptom score.

In the univariate analysis, the risk factors found to be associated with worsened dry-eye symptoms during the COVID-19 lockdown were an age up to 60 (OR 2.56; 95% CI 1.19–5.52); female (OR 2.07; 95% CI 1.32–3.26); increased VDT usage during the lockdown (OR 6.49; 95% CI 4.03–10.46); having concurrent ocular allergic diseases (OR 2.16; 95% CI 1.21–3.87); and an abnormal Thai GHQ-12 score (OR 1.93; 95% CI 1.27–2.95). DED, glaucoma, and a decreased frequency of artificial-tear use were not significantly associated. The multivariate analysis showed that being female (OR 1.86; 95% CI 1.14–3.04) and a greater VDT usage during the lockdown (OR 5.68; 95% CI 3.49–9.23) were independently associated with deteriorating dry-eye symptoms (Table 4).

**Table 4:** Univariate and multivariate analyses of risk factors that worsened dry-eye symptoms during the lockdown.

Risk factors	DED symptoms worsened	DED symptoms not worsened	*P*	OR	95% CI
	N (%)	N (%)			
Age ≤ 60 years	130 (94.2)	343 (86.4)	0.02*	2.56	1.19–5.52
DED	56 (40.6)	143 (36.0)	0.36	1.21	0.82–1.80
Female	108 (78.3)	145 (36.5)	0.001*	2.07	1.32–3.26
Increased VDT usage during the lockdown	113 (81.9)	163 (41.1)	< 0.001*	6.49	4.03–10.46
Ocular allergic disease	22 (15.9)	32 (8.1)	0.01*	2.16	1.21–3.87
Glaucoma	6 (4.3)	11 (2.8)	0.4	1.6	0.58–4.40
Abnormal Thai GHQ-12 score	49 (35.5)	88 (22.2)	0.002*	1.93	1.27–2.95
**In DED participants**	N = 56	N = 143			
Decreased frequency of artificial tear use during COVID-19 lockdown	6 (10.7)	20 (14.0)	0.64	0.72	0.27–1.91

**Risk factors**	*P*	OR	95% CI		
**Multivariate analysis**					
Age ≤ 60 years	0.11	1.959	0.86–4.47		
Female	0.012*	1.864	1.14–3.04		
Increased VDT usage during the lockdown	< 0.001*	5.676	3.49–9.23		
Ocular allergic disease	0.263	1.437	0.76–2.71		
Abnormal Thai GHQ-12 score	0.06	1.549	0.98–2.45		

* *P* < 0.05; DED, dry-eye disease; VDT, visual display terminal.

## Discussion

COVID-19 lockdowns around the world vastly altered people’s lifestyles, activities, and behaviors. To date, several waves of the COVID-19 pandemic have occurred globally, including in Thailand. Since the first wave caused the most abrupt changes in personal behaviors and mental stress, the present cross-sectional study was carried out to determine the effects of the lockdown in Thailand on dry-eye symptoms, and their potential risk factors.

Based-on the participants’ responses, 37.2% had been diagnosed with DED by an ophthalmologist. More than half of the DED participants had a mild severity, indicated by only 1 kind of medication being used and a usage frequency of less than once daily. Dry-eye symptoms were worst among the young adults, but better in the middle to older age groups. This study proved that the COVID-19 lockdown exacerbated the dry-eye symptoms of both DED patients and normal subjects aged up to 60. The correlated risk factors were being female and increased VDT usage. Viewing habits were significantly altered by the COVID-19 lockdown. There was an obvious rise in the average daily VDT usage for all purposes. The number of participants engaging in prolonged VDT usage for entertainment purposes during the lockdown was triple that during pre-lockdown. Moreover, a quarter of all participants experienced mental health problems during the lockdown. Having a mental health abnormality was also associated with having more severe dry-eye symptoms.

This community-based survey reflected the dry-eye symptoms of the overall population. It demonstrated that there was an abundance of people who suffered with dry-eye symptoms but were not seeking medical care or were unaware of the importance of DED treatment and prevention. Surprisingly, this study revealed a shift in that the younger adults reported having more severe dry-eye symptoms than the elderly respondents, especially during the lockdown (Fig. 2). Also, the prevalence of DED was relatively high in participants aged 31–40 years (51% of them had a diagnosis of DED). This result contrasted with those of other studies, which showed that DED prevalence increased with age and mostly affected women^23,33^. However, the epidemiology report of TFOS DEWS II (Tear Film and Ocular Surface Society Dry Eye Workshop II) declared that relatively high DED prevalence rates were present in younger people and school children^34^. Nowadays, the rise in VDT usage might be affecting DED prevalence^10,24^, especially during COVID-19 outbreaks when work-from-home and lockdown measures are instituted. This study identified that younger adults were exposed to VDTs longer than the elderly in both the normal and lockdown situations (Table 2) as there was a negative correlation between age and VDT usage. A rise in VDT usage was noticeably associated with worsened dry-eye symptoms, regardless of the presence of DED, which resulted in a lower DESS for the younger adults than the elderly respondents. This implies that the negative impact of VDT usage, as an external factor, could even overcome the internal risk factors of DED. Therefore, DED could eventually cause a bigger problem than we are presently experiencing. The change in lifestyles and viewing habits of the new generation could progressively increase the prevalence of DED. This would place a sizeable burden on national economies, health care systems, and workplace productivity.

The overall dry-eye symptoms were worse during the lockdown than in the normal situation. However, there was no statistical difference in the degree of reduction in the DESS of the DED and non-DED groups during the lockdown. This might be because most of our DED participants had only a mild degree of the disease. Alternatively, it might be that the potential risk factors during the lockdown (for instance, VDT usage^8,9^, lower indoor humidity^35,36^, and more mental health problems^16,17^) affected both groups equally.

Interestingly, while young to middle-aged adults suffered from a worsening of dry-eye symptoms during the lockdown, only the participants older than 60 showed no significant change. This result can be supported by the observation that during the COVID-19 lockdown, the lifestyles of mostly retired participants were not as altered as much as those of the younger ones. During both the normal and the lockdown situation, elderly Thais would typically spend most of their time indoors, away from sunlight, smoke, and wind. Even though there was an overall rise in daily VDT exposure among the elderly participants, the increment in VDT usage was still only half as much as that of the younger viewers. Further studies on behavioral changes during the lockdown of elderly people would be required to confirm this hypothesis.

In the univariate analysis, several risk factors were found to be correlated with the worsening of the dry-eye symptoms during the lockdown. However, the multivariate analysis identified only 2 independent risk factors: greater VDT usage, and the female gender. During the lockdown, people mostly stayed indoors and spent more time using digital devices^5,6^. Home confinement and limited access to public spaces led to a markedly diminished engagement in physical activities. In a recent paper, Saldanha et al.^37^ reported that the daily screen time during the pandemic doubled. The participants in that investigation also suffered with dry-eye symptoms, which eventually led to a reduction in their work efficiency^37^. The findings were consistent with that of a European survey, which reported that increased screen time was significantly associated with worsening dry-eye symptoms in DED respondents^38^. Although previous research predominantly focused on overall VDT exposure during the lockdown, our results revealed details about the purposes of the daily VDT usage. This study confirmed that the overall VDT exposure was greater during the lockdown. The prolonging of the VDT usage was chiefly attributed by our respondents to their work or study needs. Surprisingly, the number of people who binge-watched TV series, video logs, and movies was 3 times higher than normal. This might be a consequence of their limited access to other entertainment activities and public areas. Moreover, the rise in VDT usage was greatly associated with a deterioration in dry-eye symptoms. This finding is consistent with work by Uchino et al.^10^, which suggested that prolonged VDT usage shortened the tear break-up time and significantly increased the risk of DED. Other studies hypothesized that longer blinking intervals that were experienced while focusing on VDTs contributed to an acceleration of tear evaporation and eventually led to DED^10,39,40^.

Female gender is a well-known risk factor for DED. Women are disproportionately susceptible to DED, and they tend to experience more severe symptoms than men^21–23^. Sex hormones have an abundant impact on tear and lipid production as well as the immune system, which plays a major role in DED^41^. DED in females is related to their low androgen and high estrogen levels, relative to males. Therefore, as women are predisposed to having more severe dry-eye symptoms, exposure to aggravating conditions might produce worse symptoms in women than in men.

Social distancing and avoiding mass gatherings limited physical interactions between family members, friends, and colleagues. Moreover, restrictions on access to public areas like gymnasiums, fitness centers, and parks decreased the level of physical activity by the overall population. The distress caused by the COVID-19 pandemic, isolation measures, and sedentary lifestyles, contributed to an excess of mental health problems^3,6,11^. Our study found that 25.7% of the participants had developed mental health abnormalities. The prevalence was similar to those of other studies, which reported values ranging from 11 to 43.7%^11,12,42^. Furthermore, the current investigation revealed that having mental health problems correlated with having more severe dry-eye symptoms (a DESS of less than 75) during the lockdown. According to a recent European survey report, increased mental stress and poor sleep quality were listed in the top three most common causes of worsening DED symptoms during the lockdown^38^. This result was consistent with studies which had found an association between psychiatric problems and DED^15–20^. The reason is that the perception of dry-eye symptoms was influenced by several psychological traits, such as anxiety and depression^43,44^.

The study’s strength is that it provided a large-scale, community-based survey which assessed not only patients seeking medical care but also members of the general population. Moreover, the research assessed a broad range of potential risk factors for dry-eye symptoms that might be affected by the lockdown. Only a few reports revealed that the impact of a lockdown aggravated DED symptoms through excessive VDT use^37,45^.

One of the limitations of this study is that it relied on self-reported online survey, distributing the questionnaire via social media may result in sampling bias. The survey's accessibility was influenced by factors such as the use of screen-based devices and social media accounts. Another disadvantage was the lack of an objective assessment tool to determine the average daily VDT usage. As well, weekday and weekend usage might vary greatly in terms of purpose and time. This limitation should be considered during the conduct of a further study. Also, the symptoms of dry eye surveyed are nonspecific and can be attributable to other ocular surface diseases such as allergic conjunctivitis, exposure keratopathy, ocular rosacea, blepharitis and episcleritis. Finally, it might be challenging to use a question to ascertain whether there had been a previous ophthalmologic diagnosis of DED in order to assess the prevalence of the disease. This is because the criteria for the clinical diagnoses might have differed among the clinicians concerned. Unfortunately, objective evaluations of DED could not be employed for the present research due to the social distancing measures in force during the pandemic.

The lockdown measures implemented to curb the spread of the COVID-19 pandemic abruptly altered the way of living and established a new normal. The social isolation measures restricted individuals’ ability to interact with one another. This resulted in an increased usage of VDTs and frequently excessive exposure to digital devices. These behavioral changes during the lockdown deteriorated dry-eye symptoms, and they possibly led to an increase in the DED incidence.

## Data Availability

All data generated or analyzed during this study are available from the corresponding author on reasonable request.
